# A standardized combination of *Boswellia serrata* and *Terminalia chebula* extracts to improve cognition in adults with subjective memory complaints: a randomized controlled proof-of-concept study

**DOI:** 10.3389/fnut.2025.1695341

**Published:** 2025-12-08

**Authors:** Dawna Salter, Silla Vijaya Bhaskara Gupta, Bendi Sairam, Ashish Kumar Gupta, Ravishankar Maurya

**Affiliations:** 1 Clinical Research and Innovation Division, PLT Health Solutions, Morristown, NJ, United States; 2 Department of Psychiatry, Government Medical College and General Hospital, Andhra Pradesh, India; 3 Psychiatry Division, Arogya Jana Seva Kendra, Uttar Pradesh, India

**Keywords:** amyrin, Athens insomnia scale, brain-derived neurotrophic factor, cognitive function, dietary supplement, Rey’s auditory verbal learning test, sleep quality, boswellic acids

## Abstract

**Objective:**

*Boswellia serrata* (Indian frankincense) and *Terminalia chebula* (haritaki) are botanicals widely used in Indian Ayurvedic Medicine for cognitive health. This randomized, double-blind, placebo-controlled proof-of-concept study evaluated the effectiveness of LN19184, a unique, standardized blend of *B. serrata* gum resin and *T. chebula* fruit extracts, to improve cognitive function, sleep quality, and brain-derived neurotrophic factor (BDNF) levels in aging individuals.

**Methods:**

One hundred participants aged 40–65 with subjective complaints of poor memory were randomized into two groups: 300 mg of LN19184 or placebo, once daily for 120 days. At baseline and days 15, 30, 60, and 120, two neuropsychological batteries, the Rey’s Auditory Verbal Learning Test (RAVLT) and the Cambridge Neuropsychological Test (CANTAB), were used to assess cognitive function, and the Athens Insomnia Scale was used to evaluate sleep quality. Serum BDNF levels and safety parameters were also assessed.

**Results:**

LN19184 improved each measured RAVLT outcome compared to placebo. Supplementation improved proactive interference (*p* < 0.001) and recognition index (*p* = 0.004) by day 15. Learning rate, total learning, and delayed recall improved by day 60 (*p* < 0.001), and immediate recall improved by day 90 (*p* = 0.003). Compared to placebo, supplementation also improved CANTAB assessments of visual learning, processing speed, and accuracy (*p* < 0.05), sleep quality (*p* < 0.001), and serum BDNF levels (*p* < 0.001). Vital signs and safety parameters remained within normal clinical limits throughout the study.

**Conclusion:**

This pilot trial provides early empirical evidence demonstrating that a novel extract blend of *B. serrata* and *T. chebula* was well tolerated and improved cognitive function, sleep quality, and peripheral BDNF levels in aging adults with memory complaints.

**Clinical trial registration:**

Clinicaltrials.gov, identifier CTRI/2020/08/027368.

## Introduction

Cognitive decline can be a disabling condition in aging populations, limiting memory capacity and progressing from age-related cognitive decline to mild cognitive impairment (MCI) through various forms of dementia, such as Alzheimer’s Disease. As the global population continues to age, the frequency of age-related cognitive decline and dementia is expected to increase in parallel (1), presenting a growing economic, social, and health burden. The preservation of cognitive function is integral to maintaining functional abilities and independent living as one ages. As cognition declines, particularly beyond MCI, the ability to live independently decreases (2), so that cognitive decline becomes an outsized influence on the subtle declines in instrumental activities of daily living (3). To date, there are few effective pharmacologic agents to prevent or treat dementia, and interventions to manage cognitive decline are largely limited to physical exercise and cognitive training (4–7). However, epidemiological studies have consistently shown strong associations between increased consumption of plant foods and better long-term cognitive health outcomes (8, 9). Evidence continues to demonstrate that dietary factors can positively impact cognitive function (10–13) and interest in utilizing natural dietary or plant-based nutritional supplements to support cognition with aging is rising (14).

Botanical extracts derived from the *Boswellia serrata* tree (Indian frankincense) and the fruit of *Terminalia chebula* (haritaki) have long been used in Ayurvedic and other traditional medicine practices to exert antioxidant, anti-inflammatory, and cognition-supporting effects (15–18). Several lines of animal research have shown promising effects of *B. serrata* or *T. chebula* preparations on neural outcomes. *B. serrata* gum resin limited the extent of cognitive impairment in diabetic rats (19) and improved measures of spatial learning and memory that coincided with an upregulated expression of brain-derived neurotrophic factor (BDNF) in rat hippocampus (20). The anti-inflammatory and antioxidant properties of a *B. serrata* gum resin were also shown to protect dopaminergic neurons so that motor impairments were curtailed in a rodent model of Parkinson’s disease (21). Likewise, the considerable antioxidant activity of *T. chebula* provided neuroprotection and memory support in several animal models of memory impairment (22–24). Preliminary clinical research suggests cognitive benefits of boswellia or terminalia extracts may exist in human populations (25–29), but the evidence base remains limited.

LN19184 was developed as an extract blend of *T. chebula* and *B. serrata* standardized to the active constituents of gallic acid, ellagic acid, and amyrins, compounds with noted anti-inflammatory and antioxidant properties, and preclinical evidence suggesting neuroprotective and cognitive-supporting properties (30–34). When tested in a series of unpublished *in vitro* experiments, a specific 4:1 preparation of *T. chebula* fruit and *B. serrata* gum extracts consistently demonstrated dose-dependent inhibition of acetylcholinesterase (AChE) and synergistic inhibition of superoxide free radicals in human neuroblastoma cells. Efficacy, dosing, and safety were further established though dose-dependent protection from lipopolysaccharide-induced neuroinflammation and spatial learning improvement in the rodent Morris water maze (unpublished data). Based on these preclinical data, it was hypothesized that 300 mg of LN19184 could impact cognitive function in older adults with subjective memory impairment, similar to what has been shown in clinical studies investigating other herbal ingredients for memory improvements, such as *Bacopa monnieri* (35, 36), *Panax ginseng* (37), *Ginkgo biloba* (38), or *Curcuma longa* (39, 40). This pilot clinical study was designed to evaluate the effectiveness of 300 mg LN19184 supplementation daily on measures of cognitive function, as assessed by two cognitive assessment batteries: the Rey’s Auditory Verbal Learning Test (RAVLT) and the Cambridge Neuropsychological Test Automated Battery (CANTAB) over 120 days. Because there are reported associations between sleep duration and cognitive decline, measures of sleep quality were also evaluated (41). Additionally, peripheral BDNF, a biomarker of neuronal plasticity (42) and clinical safety parameters were assessed. RAVLT is a well-established measure of verbal memory that assesses recall, learning, and focus (43, 44) that has been used previously to evaluate the effects of nutritional supplements on memory in healthy individuals and those experiencing MCI (45–47). CANTAB is an automated, computerized cognitive test system validated to assess multiple cognitive domains, including executive function, memory, learning, attention, and problem-solving (48).

## Materials and methods

### Plant raw materials and extraction procedures

The *B. serrata* and *T. chebula* raw plant materials were procured from approved vendors in Madhya Pradesh and Andhra Pradesh, India, respectively. After taxonomy verification, voucher specimens of *B. serrata* (No: 6373) and *T. chebula* fruits (No: 6274) were preserved in the herbarium of the Taxonomy Division at Laila Nutra Private Limited, Vijayawada, India. *B. serrata* gum resin was extracted with aqueous ethanol at 60–85 °C. The extract was collected and filtered, and the concentrated extract was subjected to phase separation to obtain *B. serrata* nonacidic resin extract. The dried fruit of *T. chebula* was pulverized to a coarse powder and extracted with water at 65–80 °C. The extract was collected, filtered, concentrated, and dried in a vacuum dryer at 70–80 °C to obtain dried *T. chebula* fruit extract.

### Extract preparation and high-performance liquid chromatography (HPLC) analysis

The extracts of *B. serrata* gum resin and *T. chebula* fruit were blended at a 1:4 ratio to form a 90% extract blend and 10% excipient mixture. The extract blend was standardized to contain not less than 3.0% gallic acid, 1.0% ellagic acid, and 0.4% *α*- and epi-α-amyrin. Analysis was complete using an HPLC system equipped with a thermostat-controlled column oven compartment, autosampler, photodiode array detector, and Empower 3 software (Waters Corporation, Milford, MA). A known weight of LN19184 was extracted using dimethyl sulfoxide and water (50:50), followed by filtration through a 0.22 μm polyvinylidene fluoride (PVDF) filter to identify gallic acid and ellagic acid. The sample solution was analyzed using a gradient elution system consisting of solvent A (0.1% orthophosphoric acid in water; v/v) and solvent B (acetonitrile) as mobile phase with a flow rate of 0.6 mL/min using a Waters column, X Bridge C18 3.5 μm (100 × 4.6 mm), maintained at 30 °C. The run started with a mixture of 98% A and 2% B as the initial eluent and maintained an isocratic run for 9 min. Next, a linear gradient was used to reach 85% A and 15% B in 11 min, followed by isocratic elution with 85% A and 15% B for 7 min. The phytochemical marker compounds were identified by comparison of retention times with those of the reference standards. For *α*-amyrin and epi-α-amyrin identification, methanol extracted-LN19184 was filtered through a 0.22 μm PVDF filter, then analyzed using a Kinetex, Biphenyl, 2.6 μm (150 × 4.60 mm) column (Phenomenex, Torrance, CA), maintained at 30 °C. The sample was eluted for 40 min using an isocratic method with a mixture of 34% solvent A (0.1% orthophosphoric acid in water; v/v) and 66% solvent B (acetonitrile) as the mobile phase and a flow rate of 1.0 mL/min.

### Clinical study ethics approval and registration

This randomized, double-blind, placebo-controlled proof-of-concept study was conducted at two independent research organizations, in accordance with the Declaration of Helsinki and the International Conference on Harmonization guidelines on Good Clinical Practice. The Institutional Ethics Committee approved the study protocol for each study site: the by Opal Institutional Ethics Committee (Varanasi, Uttar Pradesh, India, registration number ECR/976/Inst/UP/2017/RR-20) and the Institutional Ethics Committee of the Government College & Government General Hospital (Srikakulam, Andhra Pradesh, India, registration number ECR/492/Inst/AP/2013/RR-16). This study was registered with the Clinical Trials Registry of India (Registration no. CTRI/2020/08/027368).

### Clinical study participants

Healthy male and female participants were eligible for study inclusion if they were between the ages of 40 and 65 years, had a body mass index (BMI) of 18–29 kg/m^2^, had subjective memory complaints, achieved a score of 19–30 on a screening Mini-Mental State Examination (MMSE), and had a score of 0–7 on the Hamilton depression (HAM-D) rating scale. Subjects were excluded if they had a history of any significant neurologic disease, a diagnosis of sleep apnea, a history of major depression or other psychiatric disorder, alcohol or substance use, or an abnormal screening electrocardiogram. Each volunteer was aware of the study protocol and provided written informed consent before any study-related procedures were conducted.

### Clinical study design

**Figure 1:**
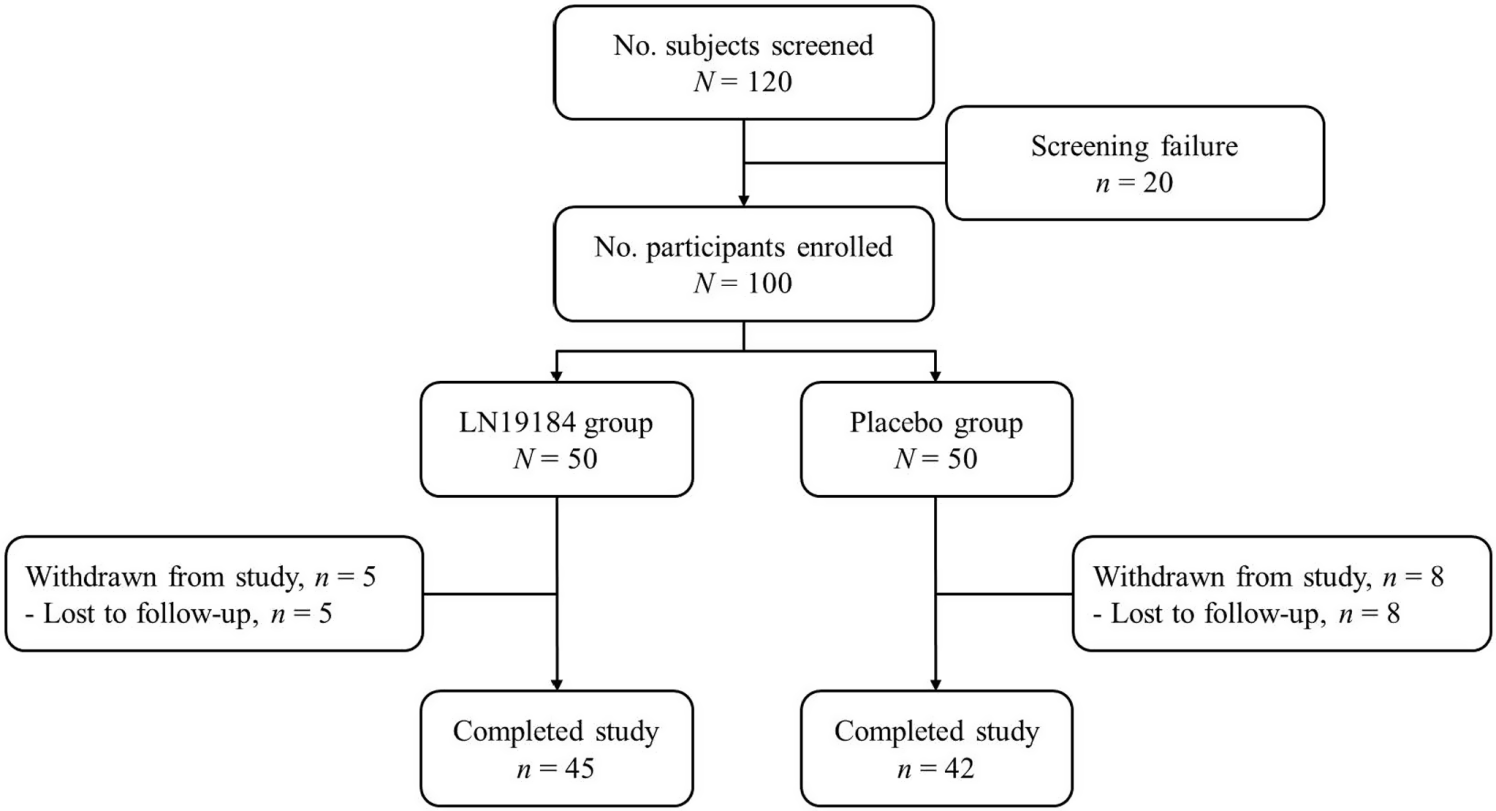
Consort diagram of participant flow through the phases of the 2-group parallel randomized blinded trial.

A total of 100 male and female subjects were enrolled and randomly allocated into one of two groups (*n* = 50) by following the randomization codes as generated by the SAS procedure PROC PLAN (SAS Institute; Cary, NC, USA) using a block design: 300 mg LN1918 or placebo. The study involved seven scheduled visits to the study sites: Screening (Day −7 to 9), Randomization /Baseline (Day 1), and Testing Days 15, 30, 60, 90, and 120. The study products, labeled by randomization code, were dispensed to subjects on study days 1, 15, 30, 60, and 90. Participants were instructed to consume one capsule daily, taken in the morning after breakfast, for 120 days. Supplement consumption compliance was monitored through daily diary recordings and the collection/counting of all unused capsules. Participants were instructed to maintain their habitual dietary intake and physical activity levels. Participants completed written 7-day diet records for one week between screening and baseline and for the last week of the study, and these diet records were evaluated for consistent eating patterns. Adverse events, tolerance, and vital signs—heart rate, blood pressure, temperature, and respiratory rate—were assessed throughout the study and recorded at each study visit. Body weights were measured with a standard scale in triplicate to the nearest 0.1 kg, and the average was recorded at each visit. Fasting blood samples were collected at screening and on Day 120. Cognitive function and sleep quality were assessed at screening, baseline, and all subsequent study site visits. All participants, investigators, and study personnel remained blinded to treatment assignment throughout the study until the data were locked. All study procedures were administered by appropriately trained and licensed study staff. The flow of the study progress is shown in the Consort diagram (Figure 1).

### Clinical study interventions

The investigational product, LN19184, contains extracts derived from *B. serrata* gum resin and *T. chebula* fruit standardized to a minimum of 3.0% gallic acid, 1.0% ellagic acid, and 0.4% amyrin. The investigational product and placebo were manufactured under a strict Good Manufacturing Process, packaged as capsules, and labeled by randomization code by Laila Nutra Private Limited (Vijayawada, India). LN19184 is commercially available as Nutricog™ from PLT Health Solutions (Morristown, NJ, USA).

### Clinical study outcome measures

The primary endpoint was a change in measures of delayed recall from Day 1 to Day 120 as assessed using RAVLT. Secondary endpoints were additional RAVLT assessments (immediate recall, total learning, learning rate, proactive interference, and recognition index), CANTAB assessments, sleep scales, and changes in peripheral BDNF levels. Safety parameters, including complete blood cell counts and blood chemistry— liver function tests, renal function tests, plasma total cholesterol, low- and high-density lipoprotein (LDL, HDL) cholesterol, triglycerides, and glucose—were assessed from fasting blood samples taken at screening and Day 120. Test parameters for urine analysis included specific gravity, pH, albumin, bile salts, bile pigment, glucose, red blood cells, and ketones.

### Rey’s auditory verbal learning test (RAVLT)

The RAVLT testing procedure followed the procedure recommended by Schmidt (44). Two 15-word lists were used to generate outputs that assessed immediate recall, delayed recall, total learning, learning rate, proactive interference, and recognition index. Briefly, the examiner read a list of 15 words (list A) at a rate of one word per second and asked the participants to recall as many words as possible. This process was repeated four additional times (five trials in total). Next, the examiner read a second list of 15 words (list B) and asked the participants to recall as many words as possible. After a two-minute delay, the participant was asked to recall words from list A without hearing the list again. This was followed by a 20-min delay during which the participants were involved in other activities. At the end of the 20 min, the participant was asked to recall as many words as possible from list A. Finally, the examiner gave the participants a list of 50 words and asked them to identify any words they recognized on list A.

### Cambridge neuropsychological test automated battery (CANTAB)

The CANTAB is a set of computerized cognitive assessments in key cognitive domains of psychomotor speed, attention, episodic memory, working memory, and executive function. At each visit, participants were given a standardized motor screening test to familiarize them with the touch screen and computer, after which they completed the 23 individual CANTAB tests evaluated in this study.

### Athens insomnia scale (AIS)

The AIS is a validated self-assessment psychometric instrument used to quantify sleep difficulty. It consists of 8 items which pertain to sleep induction, nighttime awakenings, final awakening, total duration of sleep, sleep quality, well-being, functioning capacity, and sleepiness during the day (49).

### Brain derived neurotrophic factor (BDNF)

BDNF, a marker of neuronal maintenance, plasticity, and neurogenesis, was evaluated using the Human BDNF ELISA Kit PicoKine® (Cat# EK0307, Boster Bio, Pleasanton, CA) according to the manufacturer’s instructions. The assay sensitivity of the test kit is <15 pg./mL.

### Safety

Safety parameters, including urinalysis, complete blood cell counts, and blood chemistry—liver function tests, renal function tests, plasma total cholesterol, low- and high-density lipoprotein cholesterol, triglycerides, and fasting glucose—were assessed from fasting blood samples taken at screening and day 120. Test parameters for urine analysis included specific gravity, pH, albumin, bile salts, bile pigment, glucose, red blood cells, and ketones.

### Statistical analysis

The population sample was evaluated to meet the normality requirements through the Shapiro–Wilk test and histogram analysis. A mixed factorial ANOVA with repeated measures on time was conducted. Main effects (time or treatment) and interaction (time × treatment) were evaluated after degrees of freedom were corrected by Greenhouse–Geisser when the sphericity assumption was violated. To further clarify the magnitude of the main effects, the calculation of partial effect size (ɳ^2^) was used and was defined as small, ≥ 0.02 < 0.06; moderate, ≥ 0.06 < 0.14; and large, ≥ 0.14. After significant main or interaction effects were identified, *a priori* planned pairwise comparisons at each time point were conducted after adjustment for multiple comparisons using the Bonferroni correction by using paired (intragroup) or unpaired (intergroup) Student t-tests. All effects were considered significant at *p* < 0.05. General characteristics and descriptive statistics are expressed as mean ± standard deviation (SD), whereas the estimated marginal means and calculated mean differences (MD) are presented as means ± standard error of the measurement (SE) and reported in conjunction with the 95% confidence interval (CI). The data were analyzed using SPSS Software version 29.0 (SPSS, Inc., Chicago, IL, USA).

*A priori* power analysis based on published clinical data with a natural product (47) calculated that a sample size of at least 38 participants per group would be required to achieve 85% power to detect a treatment effect in the primary efficacy variable, delayed recall, at a two-sided significance level of 0.05%. With an estimated dropout rate of 10–15%, recruitment was determined at 100 subjects. One hundred twenty participants were assessed for eligibility, and 20 failed screening due to not meeting the inclusion/exclusion criteria. After 100 subjects were randomized into 2 equal groups (*n* = 50), 13 subjects dropped out due to scheduling difficulties or protocol violations during the study. In total, 87 subjects completed the study: LN19184, *n* = 45; Placebo, *n* = 42. In some instances, the computerized CANTAB system did not capture responses fully, resulting in incomplete datasets for 7 subjects: 4 in the active group and 3 in the placebo group. These subjects were removed from the analysis, leaving 80 subjects with completed CANTAB evaluations: LN19184, *n* = 41; Placebo, *n* = 39.

## Results

### HPLC

**Figure 2:**
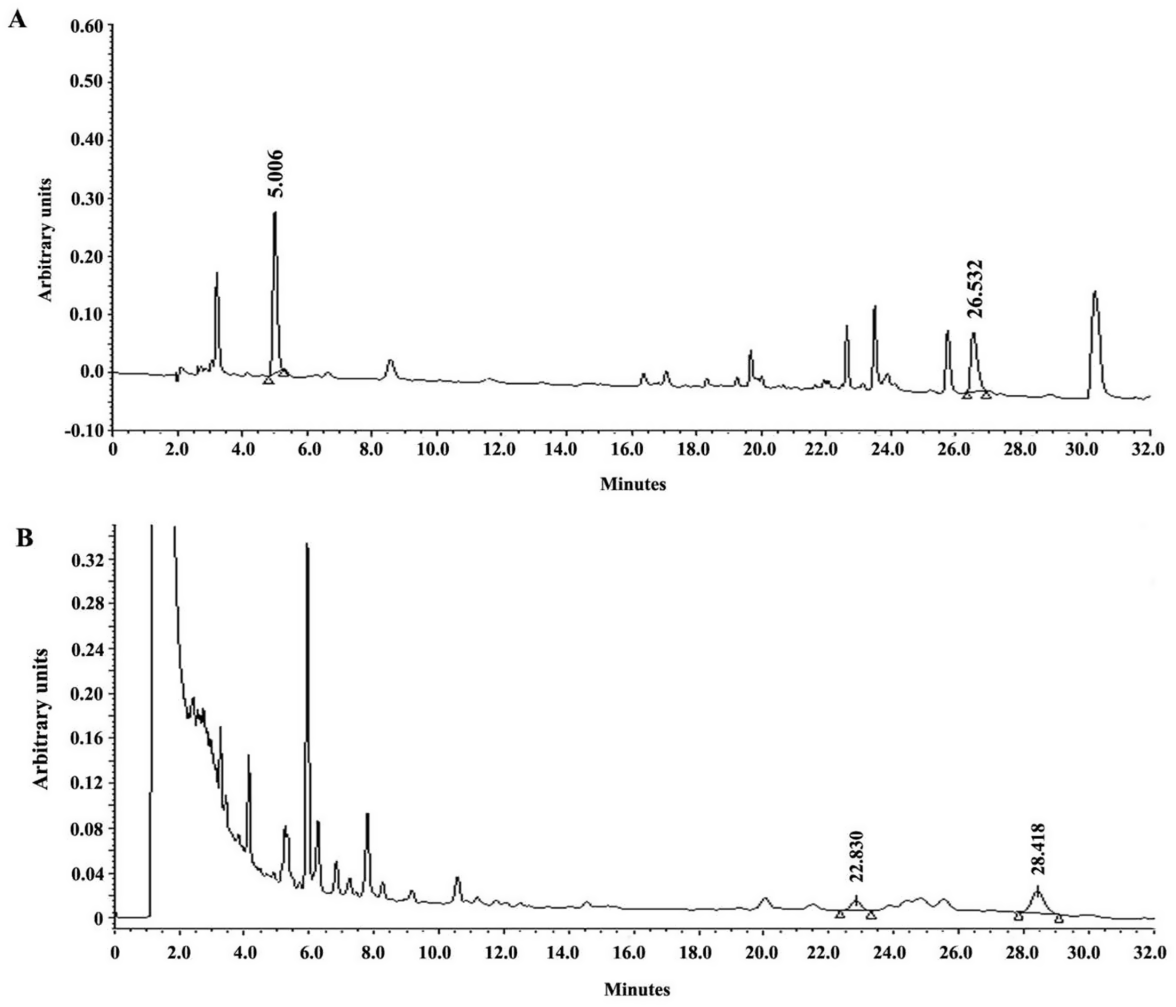
HPLC chromatograms of LN19184. A representative elution profile at 271 nm shows **(A)** the peaks eluted at 5.006 and 26.532 min representing gallic acid and ellagic acid, respectively, and **(B)** the peaks eluted at 22.830 and 28.418 min representing a-amyrin and epi-a-amyrin, respectively.

Figures 2A,B depict a typical HPLC chromatogram of LN19184. Figure 2A illustrates the chromatogram profile at 271 nm with the elution of two peaks, gallic acid at 5.006 and ellagic acid at 26.532 min. Figure 2B illustrates a chromatographic profile at 210 nm demonstrating two peaks eluted at 22.830 and 28.418 min, identified as *α*-amyrin and epi-α-amyrin, respectively.

### Clinical study participant characteristics, adverse events, and compliance

Demographic and background characteristics were similar between the groups (Table 1). Compliance with treatment consumption was calculated to be greater than 80% for all participants who completed the study, meeting the predetermined weekly compliance criteria. No serious adverse events were reported during the study. Overall, five participants experienced minor adverse events, three in the placebo group and two in the treatment group. Specific adverse events within the placebo group consisted of one subject complaining of bloating, one experiencing an episode of vomiting, and one reporting symptoms of minor muscular pain. In the treatment group, one participant experienced bloating, and one complained about itching. These adverse events were self-limiting and resolved fully during the study. Safety parameters and vital signs were within normal clinical ranges at baseline, and there were no clinically significant changes in either subject group. Hematological and biochemical parameters are reported in Supplementary Tables S1, S2.

**Table 1:** Participant demographic and background characteristics.

Parameters	PLA	LN19184	*P* score
N	50	50	
Sex (M/F)	46/4	41/9	
Age (Years)	48.6 **±** 8.3	49.2 **±** 8.0	0.696
Body Weight (kg)	67.6 ± 5.7	66.7 ± 6.7	0.499
BMI (kg/m^2^)	24.4 ± 2.1	24.0 ± 2.2	0.307
HAM-D score	4.5 **±** 1.5	4.6 **±** 1.5	0.895
MMSE score	24.4 **±** 3.0	24.1 **±** 2.6	0.639

Values are means ± standard deviation of the randomized population at study initiation. BMI (body mass index), HAM-D (Hamilton depression rating scale), MMSE (mini-mental state examination).

### RAVLT delayed recall

Changes in RAVLT scores over time are shown in Table 2. Analysis of delayed recall demonstrated main effects of time and treatment with large effect sizes (*p* < 0.001, ɳ^2^ = 0.787; *p* < 0.001, ɳ^2^ = 0.155, respectively). There was also a time × treatment interaction with a large effect size (*p* < 0.001, ɳ^2^ = 0.405). Both groups improved in measures of delayed recall compared to their respective baseline measures. The placebo group improved delayed recall on Day 30 through Day 120 (Day 30, *p* = 0.009; Days 60/90/120, *p* < 0.001), and the LN19184 group improved on Day 15 through Day 120 (*p* < 0.001 for all). Delayed recall was significantly better in the LN19184 group compared to the placebo group starting on Day 60 and continuing through Day 120 (*p* < 0.001 for all; Table 2).

**Table 2:** Effect, effect size, within- and between-group comparisons of RAVLT subtests.

Parameter	Group	Evaluation days						Main effect *p*-value Effect size (ɳ^2^)
		Day 1 Baseline	Day 15	Day 30	Day 60	Day 90	Day 120	
Delayed recall	LN19184 ± SD	4.7 ± 0.8	5.8 ± 0.9*	6.6 ± 1.2*	8.0 ± 1.1*	9.2 ± 1.1*	10.8 ± 1.0*	(t) *p* < 0.001^#^ ɳ^2^ = 0.787 (L) (trt) *p* < 0.001^#^ ɳ^2^ = 0.155 (L) (int) *p* < 0.001^#^ ɳ^2^ = 0.405 (L)
	PLA ± SD	5.7 ± 1.8	6.0 ± 1.3	6.4 ± 1.4*	6.8 ± 1.3*	7.3 ± 1.1*	8.2 ± 1.3*	
	MD ± SE 95% CI	−1.1 ± 0.3**^** −1.7, − 0.5	−0.2 ± 0.2 −0.7, 0.3	0.3 ± 0.3 −2.8, 0.8	1.2 ± 0.3 **^** 0.6, 1.7	1.9 ± 0.2 **^** 1.5, 2.4	2.6 ± 0.2 **^** 2.1, 3.1	
Immediate recall	LN19184 ± SD	5.2 ± 1.0	5.9 ± 0.8*	6.1 ± 1.0*	6.5 ± 1.0*	6.7 ± 0.9*	7.3 ± 0.9*	(t) *p* < 0.001^#^ ɳ^2^ = 0.405 (L) (trt) *P* < 0.015^#^ ɳ^2^ = 0.067(M) (int) *p* < 0.001^#^ ɳ^2^ = 0.074(M)
	PLA ± SD	5.4 ± 1.0	5.6 ± 1.0	5.6 ± 1.1	6.1 ± 1.2*	6.1 ± 1.1*	6.3 ± 1.2*	
	MD ± SE 95% CI	−0.2 ± 0.2 −0.6, 0.3	0.3 ± 0.2 −0.1, 0.7	0.4 ± 0.2 −0.1, 0.9	0.4 ± 0.2 −0.0, 0.9	0.6 ± 0.2**^** 0.2, 1.1	1.0 ± 0.2**^** 0.5, 1.4	
Total learning	LN19184 ± SD	30.6 ± 4.0	34.0 ± 3.4*	35.9 ± 3.5*	40.0 ± 3.8*	43.3 ± 4.2*	47.9 ± 4.4*	(t) *p* < 0.001^#^ ɳ^2^ = 0.735 (L) (trt) *p* = 0.007^#^ ɳ^2^ = 0.081(M) (int) *p* < 0.001^#^ ɳ^2^ = 0.316 (L)
	PLA ± SD	32.5 ± 6.5	34.3 ± 6.4*	35.0 ± 6.1*	36.8 ± 5.6*	37.5 ± 5.9*	40.2 ± 6.2*	
	MD ± SE 95% CI	−1.8 ± 1.1 −4.1, 0.4	−0.3 ± 1.1 −2.4, 1.9	0.9 ± 1.1 −1.2, 3.0	3.2 ± 1.0**^** 1.2, 5.3	5.8 ± 1.1**^** 3.7, 8.0	7.8 ± 1.1**^** 5.5, 10.0	
Learning rate	LN19184 ± SD	1.8 ± 0.8	2.0 ± 0.9	2.4 ± 1.2*	2.9 ± 1.5*	4.2 ± 1.2*	4.9 ± 1.5*	(t) *p* < 0.001^#^ ɳ^2^ = 0.425 (L) (trt) *p* = 0.134 ɳ^2^ = 0.026 (S) (int) *p* < 0.001^#^ ɳ^2^ = 0.188 (L)
	PLA ± SD	2.4 ± 1.5	2.8 ± 1.5	2.9 ± 1.7	1.3 ± 1.8*	3.2 ± 1.5*	3.8 ± 1.5*	
	MD ± SE 95% CI	−0.6 ± 0.3**^** −1.1, −0.1	−0.8 ± 0.3**^** −1.3, −0.3	−0.4 ± 0.3 −1.1, 0.2	1.6 ± 0.4**^** 0.9, 2.3	1.0 ± 0.3**^** 0.4, 1.6	1.1 ± 0.3**^** 0.5, 1.8	
Proactive interference	LN19184 ± SD	0.4 ± 1.2	−0.1 ± 1.0	−0.9 ± 0.9*	−2.0 ± 1.2*	−2.8 ± 1.2*	−3.6 ± 1.5*	(t) *P* < 0.001^#^ ɳ^2^ = 0.461 (L) (trt) *p* < 0.001^#^ ɳ^2^ = 0.582 (L) (int) *p* < 0.001^#^ ɳ^2^ = 0.283 (L)
	PLA ± SD	0.7 ± 1.2	0.7 ± 1.0	0.6 ± 1.3	0.4 ± 1.5	0.1 ± 1.4	0.0 ± 1.4	
	MD ± SE 95% CI	−0.4 ± 0.2 −0.9, 0.2	−0.8 ± 0.2**^** −1.2, −0.3	−1.5 ± 0.3**^** −2.0, −1.0	−2.4 ± 0.3**^** −3.0, −1.8	−2.9 ± 0.3**^** −3.5, −2.4	−3.7 ± 0.3**^** −4.3, −3.0	
Recognition index	LN19184 ± SD	5.3 ± 1.0	6.2 ± 1.0*	7.2 ± 1.2*	8.3 ± 1.0*	9.5 ± 1.1*	11.0 ± 1.4*	(t) *p* < 0.001^#^ ɳ^2^ = 0.662 (L) (trt) *p* < 0.001^#^ ɳ^2^ = 0.757 (L) (int) *p* < 0.001^#^ ɳ^2^ = 0.618 (L)
	PLA ± SD	5.0 ± 0.9	5.4 ± 1.1	5.2 ± 1.0	5.5 ± 1.0*	5.2 ± 0.9	5.4 ± 0.9	
	MD ± SE 95% CI	0.3 ± 0.2 −0.1, 0.7	0.8 ± 0.2**^** 0.3, 1.2	2.0 ± 0.2**^** 1.5, 2.5	2.7 ± 0.2**^** 2.3, 3.2	4.3 ± 0.2**^** 3.8, 4.7	5.5 ± 0.3**^** 5.0, 6.1	

Data presented as mean ± standard deviation (SD) or mean difference (MD) ± standard error (SE) and 95% confidence interval (95% CI). Significance is considered *p* < 0.05 after mixed factorial repeated measure ANOVA adjusted with Bonferroni correction for multiple comparisons * Indicates within-group significance (vs. baseline), ^ indicates significant difference between group means (LN19184 vs. placebo), # indicates significant main effect of time (t), treatment (trt,) or time x treatment interaction (int). Partial effect size (ɳ^2^) is defined as small (S), ≥ 0.02 < 0.06; moderate (M), ≥ 0.06 < 0.14; and large, ≥ 0.14 (L). LN19184 *n* = 45, PLA (Placebo) *n* = 42. RAVLT (Rey’s Auditory Verbal Learning Test).

### RAVLT immediate recall

Analysis of the RAVLT measure of immediate recall revealed main effects of time and treatment, with large and moderate effect sizes (*p* < 0.001, ɳ^2^ = 0.405; *p* < 0.015, ɳ^2^ = 0.067, respectively). The time × treatment interaction was also significant with a moderate effect size (*p* < 0.001, ɳ^2^ = 0.074; Table 2). Participants in both groups improved their correct answers in the RAVLT immediate recall test over time compared to baseline measures. The placebo group improved immediate recall on Days 60, 90, and 120, while participants in the LN19184 group improved on Days 15, 30, 60, 90, and 120 (*p* < 0.001 for all). Between-group comparisons demonstrated significant differences in immediate recall, with LN19184 scoring higher than the placebo group on Day 90 (*p* = 0.003) and Day 120 (*p* < 0.001).

### RAVLT total learning score

The RAVLT total learning score demonstrated main effects of time and treatment with large and moderate effect sizes (*p* < 0.001, ɳ^2^ = 0.735; *p* = 0.007, ɳ^2^ = 0.081, respectively). There was also a time × treatment interaction with a large effect size (*p* < 0.001; ɳ^2^ = 0.316, Table 2). There were significant improvements from baseline in both the placebo and LN19184 groups from Day 15 through Day 120 (placebo, Days 15/30/60/90/120, *p* < 0.001; LN19184, Days 15/30/60/90/120, *p* < 0.001 for all). The LN19184 group scored significantly better in total learning than placebo from Day 60 through Day 120 (Day 60, *p* = 0.002; Days 90/120, *p* < 0.001).

### RAVLT learning rate

The RAVLT learning rate demonstrated a main effect of time and a time × treatment interaction, both with large effect sizes (*p* < 0.001, ɳ^2^ = 0.425; *p* < 0.001, ɳ^2^ = 0.188, respectively). The main effect of treatment was not significant (*p* = 0.134, Table 2). Compared to the baseline score, the learning rate first decreased on Day 60 (*p* = 0.005) but then increased on Day 90 (*p* = 0.009) and Day 120 (*p* < 0.001) in the placebo group. In contrast, the LN19184 group demonstrated significant increases over baseline from Day 30 through Day 120 in the LN19184 group (Day 30, *p* = 0.011; Day 60, *p* = 0.008; Days 90/120, *p* < 0.001). Between-group comparisons indicated the placebo group had a higher learning rate compared to the LN19184 group at baseline and Day 15, but the learning rate accelerated with supplementation so that scores were significantly higher (*p* < 0.001) than placebo at Days 60, 90, and 120.

### RAVLT proactive interference

The analysis of the RAVLT proactive interference scores demonstrated significant main effects of time and treatment, and a time × treatment interaction with large effect sizes (time: *p* < 0.001, ɳ^2^ = 0.461; treatment: *p* < 0.001, ɳ^2^ = 0.582; interaction: *p* < 0.001, ɳ^2^ = 0.283; Table 2). There were no significant changes to the RAVLT proactive interference scores in the placebo group over time, but supplementation with LN19184 significantly decreased these scores compared to baseline at all timepoints, Day 30 through Day 120 (*p* < 0.001 for all). Between-group comparisons demonstrated that the proactive interference score was significantly lower with LN19184 than with placebo at all measured time points from Day 15 through Day 120 (*p* < 0.001 for all).

### RAVLT recognition memory

Analysis of the RAVLT recognition memory scores demonstrated significant main effects of time and treatment, and a time × treatment interaction with large effect sizes (time: *p* < 0.001, ɳ^2^ = 0.662; treatment: *p* < 0.001, ɳ^2^ = 0.757; interaction: *p* < 0.001, ɳ^2^ = 0.618; Table 2). Within-group comparisons indicated there was no change over time in the number of correct responses for the RAVLT recognition index in the placebo group; however, supplementation of LN19184 significantly increased the number of correct responses from baseline at all measured time points (Day 15, *p* = 0.004; Days 30/60/90/120, *p* < 0.001). Between-group comparisons indicated recognition index was significantly better with LN19184 than placebo at each time point measured (*p* < 0.001 for all).

### CANTAB processing speed: rapid visual processing median latency (RVIPMDL)

**Figure 3:**
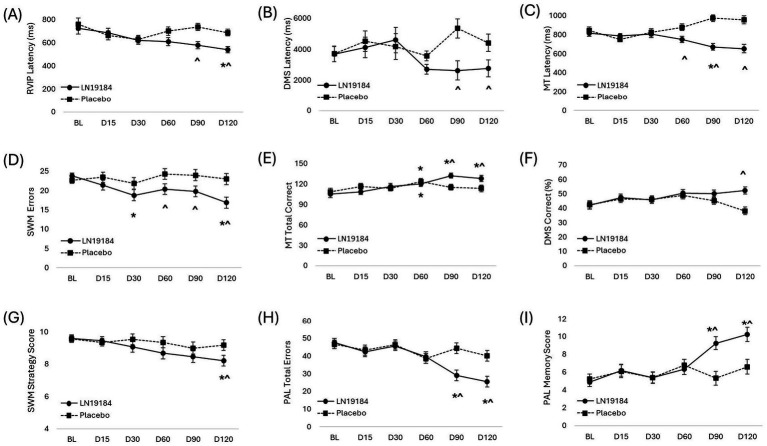
Changes in selected CANTAB test scores (Cambridge neuropsychological test automated battery). Measures of visual processing speed **(A)** RVIP latency (Rapid visual processing median latency), **(B)** DMS latency (Delayed match to sample median latency), and **(C)** MT latency (Multitasking median latency); measurements of visual processing accuracy **(D)** SWM errors (Spatial working memory errors), **(E)** MT total correct (Multitasking total correct), and **(F)** DMS Correct % (Delayed match to sample percent correct); measures of decision making and learning **(G)** SWM strategy (Spatial working memory strategy), **(H)** PAL total errors (Paired associates learning total errors), **(I)** PAL memory (Paired associates learning memory score). Data are presented as mean ± SE. Significance is considered *p* < 0.05 after mixed factorial repeated measure ANOVA adjusted with Bonferroni correction for multiple comparisons. *Indicates within-group significance (vs. baseline), ^indicates significant difference between group means (LN19184 vs. placebo). BL (baseline), D (day of testing), LN19184 (*n* = 41), Placebo (*n* = 39).

Changes in CANTAB scores over time are shown in Figure 3 and Supplementary Tables S3 and S4. Analysis of RVIP latency scores indicated significant main effects of time and treatment, both with small effect sizes (*p* = 0.001, ɳ^2^ = 0.053; *p* = 0.040, ɳ^2^ = 0.053, respectively). The time × treatment interaction was also significant with a small effect size (*p* = 0.014, ɳ^2^ = 0.036). Within-group comparisons indicated that RVIPMDL improved compared to baseline on Day 120 in the LN19184 group (*p* = 0.004), but there was no significant improvement over time in the placebo group. Between-group comparisons indicated that visual processing latency was significantly better with supplementation of LN19184 compared to placebo on Day 90 and Day 120 (*p* < 0.001 for both; Figure 3A, Supplementary Table S3).

### CANTAB processing speed: delayed match to sample median latency (DMSMDL)

Analysis of the DMSMDL demonstrated there was a significant time × treatment interaction with a small effect size (*p* = 0.036, ɳ^2^ = 0.033); however, the main effects of time and treatment were not significant (*p* = 0.131 and *p* = 0.122, respectively). Scores did not change significantly from baseline over time in either group, but the latency was lower in the LN19184 group compared to placebo at Day 90 (*p* = 0.003) and Day 120 (*p* = 0.035, Figure 3B, Supplementary Table S3).

### CANTAB processing speed: multitasking median latency (MTMDL)

Analysis of MTMDL demonstrated a main effect of treatment with a large effect size (*p* < 0.001, ɳ^2^ = 0.183) and an interaction between treatment and time with a moderately large effect size (*p* < 0.001, ɳ^2^ = 0.124). The main effect of time was not significant (*p* = 0.444). After 90 days of supplementation, MTMDL improved compared to the baseline score (*p* = 0.035), while after 120 days, the score trended toward but did not reach significance (*p* = 0.074). In contrast, the latency scores did not significantly change from their baseline values in the placebo group. Between-group comparisons indicated the MTMDL scores were better with daily supplementation of LN19184 compared to placebo on Days 60, 90, and 120 (*p* = 0.012, *p* < 0.001, and *p* < 0.001, respectively; Figure 3C; Supplementary Table S3).

### CANTAB processing accuracy: spatial working memory errors (SWME)

Supplementation affected scores of SWME as illustrated by significant main effects of time and treatment with small to moderate effect sizes (*p* = 0.018, ɳ^2^ = 0.034; and *p* = 0.015, ɳ^2^ = 0.074, respectively). The time × treatment interaction was also significant with a small effect size (*p* = 0.034; ɳ^2^ = 0.030). Within-group comparisons indicated errors were significantly decreased compared to baseline scores at Day 30 and Day 120 (*p* = 0.021 and *p* < 0.001, respectively) in the LN184 group, but the error score did not change compared to baseline in the placebo group at any measured time point. Between-group comparisons indicated that daily LN19184 supplementation resulted in better SWME scores compared to placebo on Days 60, 90, and 120 (*p* = 0.049, *p* = 0.042, and *p* = 0.004, respectively; Figure 3D; Supplementary Table S3).

### CANTAB processing accuracy: multitasking total correct score (MTTC)

For the MTTC score, there was a main effect of time and a time × treatment interaction with small to moderate effect sizes for both (*p* < 0.001, ɳ^2^ = 0.090 and *p* < 0.001, ɳ^2^ = 0.058, respectively). The main effect of treatment was not significant (*p* = 0.454). Within-group comparisons revealed that supplementation improved the total correct score while multitasking compared to baseline on Days 60 (*p* = 0.033), 90 (*p <* 0.001), and 120 (*p* < 0.001); while the placebo group only showed improvement over baseline scores on Day 60 (*p* = 0.047). Supplementation with LN19184 resulted in significantly better MTTC scores compared to the placebo group on Days 90 and 120 (*p* = 0.003 and *p* = 0.036, Figure 3E; Supplementary Table S3).

### CANTAB processing accuracy: delayed match to sample percent correct (DMSPC)

Daily supplementation with LN19184 improved accuracy on the DMSPC test over time, as shown by a significant time × treatment interaction with a small effect size (*p* = 0.021, ɳ^2^ = 0.035). Both the main effects of time and treatment trended toward but did not attain the threshold for significance (*p* < 0.051, *p* < 0.081). Over time, there were no significant changes for either group compared to their respective baseline scores. However, between-group comparisons demonstrated supplementation improved accuracy on the DMSPC scores compared to placebo on Day 120 (*p <* 0.001, Figure 3F; Supplementary Table S3).

### CANTAB decision making and learning: spatial working memory strategy (SWMS)

For the CANTAB SWMS test, the main effects of time and treatment were significant (*p* = 0.022 and *p* = 0.031, respectively), and small effect sizes for both (ɳ^2^ = 0.035 and ɳ^2^ = 0.058, respectively). The time × treatment interaction was insignificant (*p* = 0.475). Supplementation with LN19184 improved the strategy score at Day 120 compared to the baseline score (*p* < 0.012); there were no significant changes versus baseline for the placebo group. Between-group comparisons indicated supplementation improved the SWMS scores compared to placebo at Day 120. (*p* = 0.049, Figure 3G; Supplementary Table S3).

### CANTAB decision making and learning: paired associates learning total errors (PALTE)

Analysis of the CANTAB PALTE scores revealed significant main effects of time and treatment, as well as a time × treatment interaction with small to moderate-sized effects for both tests. (time: *p* < 0.001, ɳ^2^ = 0.111; treatment: *p* = 0.029, ɳ^2^ = 0.060; interaction: *p* < 0.001, ɳ^2^ = 0.058). Supplementation improved the score for errors on the PAL test, as scores at Days 90 and 120 were significantly decreased compared to baseline (*p* < 0.001 for both). In contrast, the placebo group showed no significant changes over time. Between-group comparisons showed that PALTE was significantly lower with LN19184 supplementation compared to placebo on Days 90 and 120 (*p* < 0.001 for both, Figure 3H; Supplementary Table S3).

### CANTAB decision making and learning: paired associates learning first attempt memory score (PALFAMS)

Analysis of PALFAMS demonstrated supplementation affected visual–spatial memory and learning as illustrated by significant main effects of time and treatment with moderate effect sizes (*p* < 0.001, ɳ^2^ = 0.085; and *p* = 0.014, ɳ^2^ = 0.074, respectively). The time × treatment interaction was also significant with a moderate effect size (*p* < 0.001; ɳ^2^ = 0.061). Within-group comparisons revealed that supplementation improved the memory score compared to baseline on Days 90 and 120 (*p* < 0.001 for both), while the placebo group did not improve significantly over baseline scores at any measured time point. Between-group comparisons showed supplementation with LN19184 significantly improved the PALFAMS compared to the placebo group on Days 90 (*p* < 0.001) and 120 (*p* = 0.002, Figure 3I; Supplementary Table S3).

### Sleep quality

**Figure 4:**
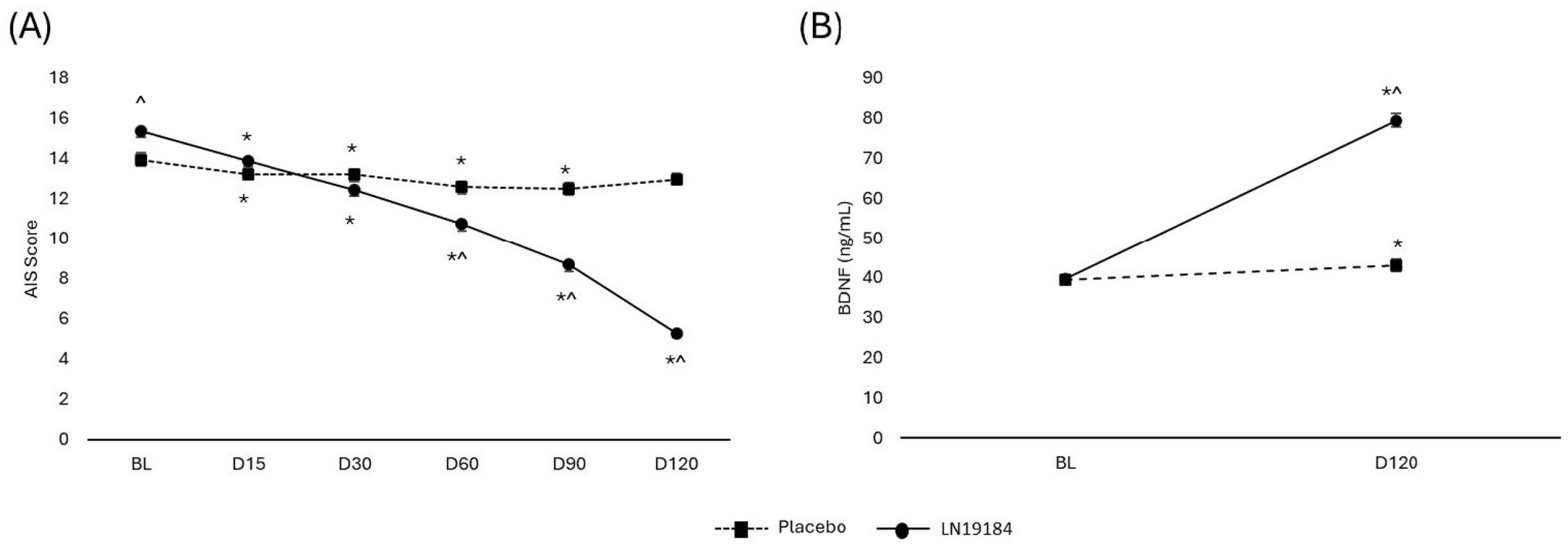
Changes in subjective sleep quality and measured serum biomarker. **(A)** AIS (Athens insomnia index) scores, **(B)** Peripheral BDNF (Brain derived neurotrophic factor). Data are presented as mean + SE. Significance is considered *p* < 0.05 after mixed factorial repeated measure ANOVA adjusted with Bonferroni correction for multiple comparisons. *Indicates within-group significance (vs. baseline), ^indicates significant difference between group means (LN19184 vs. placebo). BL (baseline), D (day of testing), LN19184 (*n* = 45), Placebo (*n* = 42).

Analysis of the AIS demonstrated main effects of time and treatment, as well as a time × treatment interaction, all with large effect sizes (time: *p* < 0.001, ɳ^2^ = 0.757; treatment: *p* < 0.001, ɳ^2^ = 0.265; interaction: *p* < 0.001, ɳ^2^ = 0.679). Within-group comparisons illustrated that sleep quality improved significantly from respective baseline scores, regardless of group assignment. However, between-group comparisons revealed that supplementation improved insomnia scores more than placebo. The LN19184 group had significantly lower insomnia scores compared to the placebo at days 60, 90 and 120 (*p* < 0.001 for all, Figure 4A).

### Serum BDNF

Analysis of serum BDNF levels revealed a main effect of time, a main effect of treatment, and a time × treatment interaction with large effect sizes (*p* < 0.001, ɳ^2^ = 0.784; *p* < 0.001, ɳ^2^ = 0.696; and *p* < 0.001, ɳ^2^ = 0.715, respectively). BDNF levels in the placebo group demonstrated an 8.5% increase over 120 days, increasing from a baseline of 39.3 ± 5.3 ng/mL to a final value of 43.0 ± 12.0 ng/mL (*p* = 0.043). In contrast, the BDNF levels increased 99.9% in the LN19184 group, increasing from a baseline value of 39.8 ± 5.9 ng/mL to a final value of 79.5 ± 8.9 ng/mL (*p* < 0.001). Between group comparisons indicated BDNF levels were significantly higher in the LN19184 than placebo at the end of the study (*p* < 0.001, Figure 4B).

## Discussion

This randomized proof-of-concept trial demonstrated that supplementing 300 mg of LN19184 for 120 days improved measures of verbal and visual memory, cognitive processing, and learning capacity compared to a placebo. Cognitive impairment presents a significant challenge to public health due to its increasing prevalence as the global population ages (1). To date, there are few effective treatments to delay or reverse cognitive decline aside from exercise and cognitive training (4, 5, 7), but a growing body of research suggests that plant-based diets or diets enriched with bioactive plant-based components can benefit cognitive function (8, 9, 50), possibly by decreasing inflammation, improving neurogenesis, and improving neuronal and synaptic connectivity (51–53).

LN19184 is a novel, standardized blend composed of extracts derived from *T. chebula* and *B. serrata*, two botanicals traditionally used to support intellect, memory, mental clarity, and focus (15, 17, 18). In the present study, RAVLT outcomes improved for all subjects relative to their baseline scores, regardless of the treatment group. The improvement in scores over time in all subjects was not surprising, as the RAVLT test was administered five separate times during the 4-month study. Repeated exposure to the RAVLT battery is well known to provide opportunities for rehearsal and practice, leading to increased recall over time (54). However, those randomized to receive daily LN19184 supplementation consistently demonstrated RAVLT outcomes that were greater than those of the placebo group. LN19184 improved measures of recognition index and proactive interference over placebo as early as Day 15, while delayed recall, total learning, and learning rate improved by Day 60, and immediate recall scores improved by Day 90. Better delayed recall and recognition index measures indicate LN19184 promoted stronger long-term verbal memory, recognition memory, and episodic memory. The higher total learning and learning rate scores with LN19184 suggest an improved learning and acquisition of new information. Supplementation also improved performance on immediate recall and proactive interference measures, suggesting more effective attention shifting, working memory, and learning strategies in this study’s population of aging individuals. The substantial gains in RAVLT outcome measures when compared to placebo indicate that LN19184 supplementation may help mitigate some of the detrimental effects of aging on brain function.

Daily LN19184 supplementation also impacted visual learning and memory, as 16 of the 23 (~70%) administered CANTAB tests demonstrated a statistically significant treatment or interaction effect in this 120-day study. Supplemented subjects showed improvements in both processing speed and accuracy despite the tendency for accuracy to be sacrificed when individuals are tested for speed in visual processing tasks, a well-established concept in cognitive research referred to as a “speed-accuracy tradeoff” (55). CANTAB tests evaluating visual processing speed consistently improved with supplementation, as latencies for RVIP and DMS decreased by day 90, and the latency for MT decreased by day 60, compared to the placebo group’s measured scores. LN19184 also improved scores for CANTAB tests assessing visual processing accuracy. Compared to placebo, LN1918 improved the percentage of correct answers on the DMS test by day 120, increased accuracy on the MT test by day 90, and decreased the number of errors on the SWM by day 60. In addition to visual accuracy, visual episodic memory and learning also improved with LN19184 by day 90. When compared to placebo, supplemented subjects improved their ability to remember the location of abstract patterns in boxes on the first try (PALFAMS), and they made fewer errors across all stages of the test (PALTE). LN19184 improved performance on the SWM strategy test with a score significantly lower than placebo by the end of the study. The reduced score indicated that supplemented subjects utilized a more consistent and effective sequence when completing the SWM task, indicating a more effective use of working memory and executive function. When viewed together, the improvements in CANTAB test outcomes suggest that subjects were better able to encode and recall memory, accelerate their learning curve, and improve executive functions with daily supplementation.

The improvement in cognition with LN1918 supplementation shown in this study is consistent with effects demonstrated by other natural, ethnomedicinal plant-based compounds that have been clinically studied for cognitive-enhancing or protective properties. Various forms of curcumin, a polyphenolic compound derived from the Indian spice turmeric, have been shown to improve long-term memory retrieval, working memory, and attention in healthy, older subjects (39, 40, 56). Ginseng (*Panax*), containing triterpenoid saponin ginsenosides, has been shown to improve performance on word recall and working memory in healthy adults (37). *Bacopa monniera* (Brahmi) is a plant used in Ayurvedic medicine to benefit memory and cognition. *B. monniera,* rich in triterpenoid saponin compounds, has been shown to support cognitive function in several clinical trials (35, 36, 57, 58). In addition, *Ginkgo biloba* leaf extracts standardized to triterpene ginkgolides and bilobalide have been widely tested in clinical trials evaluating cognitive impairment and cerebral dysfunction (38). Pentacyclic triterpenoid boswellic acids and their precursor, amyrins, are natural constituents of LN19184 with well-defined anti-inflammatory and antioxidant properties (15, 16). These boswellic triterpenoids have been shown to protect against neurotoxicity, neuroinflammation, and memory loss in numerous preclinical animal models of memory impairment (19, 21, 33, 59–62). Further, in several of these models, the boswellic compounds were shown to rescue memory impairment through the inhibition of AChE (32, 33, 59, 61), observations that are in line with *in vitro* research showing boswellic triterpenoids selectively dock to AChE (63) and exhibit considerable AChE inhibition (64). AChE breaks down acetylcholine, the key neurotransmitter involved in learning and memory. Elevated levels of AChE result in depleted levels of acetylcholine at the synaptic cleft, a phenomenon implicated in the pathophysiology of memory loss observed in neurodegenerative diseases (65). In the present study, LN19184, being naturally rich in triterpenoid compounds, may have influenced cognitive processing by inhibiting AChE and improving acetylcholine transmission. LN19184 also contains ellagic and gallic acids, which have been documented in preclinical research to show neuroprotective and cognition-enhancing potential (30, 31, 34).

LN19184 may also have improved cognitive function in the present study through changes to BDNF production or circulation. Central BDNF has been directly linked to learning and memory, as it is involved in mediating the plastic changes that are necessary for the processes of spatial and recognition memory (66, 67). Levels of circulating BDNF decrease as a person ages, which is associated with hippocampal shrinkage and reduced memory performance (68). Studies in rats and pigs have demonstrated a correlation between peripheral and brain BDNF levels (69), although this association cannot be confirmed in humans, as proven techniques to measure BDNF levels in human brain tissue remain elusive. Nevertheless, studies consistently show that improved levels of serum BDNF in adults coincide with measurable cognitive improvements (7, 23, 70, 71). Thus, interventions that can increase circulating BDNF may be beneficial for brain health and memory processes. The present study showed that after 120 days of LN19184 supplementation, serum BDNF levels were both higher than their baseline levels and higher than those BDNF levels measured in participants who received a placebo. A possible mechanism for the improvements in long-term memory that are apparent with LN19184 supplementation may be that the increased levels of BDNF are contributing to structural rearrangement and strengthening of synapses in the brain (72), as BDNF has a key role in neurogenesis, synaptic plasticity, and neuronal survival (72–74). However, the study is limited because BDNF was measured only at baseline and Day 120, making it difficult to assess if the increases in measured BDNF levels coincided with subsequent cognitive improvement. Future research on LN19184 would benefit from more frequent assessments of BDNF alongside repeated cognitive testing.

Improving sleep dynamics may have also contributed to cognitive improvements observed in this study, as LN19184 supplementation significantly decreased insomnia scores over time compared to baseline and placebo measures. Several studies have shown that people with poor sleep quality have an accelerated rate of cognitive decline or poor memory performance (41, 75). The mechanism by which LN19184 impacts sleep dynamics is unknown, but it may be related to the increased BDNF, as low levels of peripheral BDNF have been associated with insomnia (76). Alternatively, *β*-amyrin was shown to increase brain gamma-aminobutyric (GABA) levels and improve sleep quality in rats (77), suggesting that LN19184 may enhance the quality of sleep through an amyrin-dependent increase in GABA.

Overall, these findings suggest that daily supplementation with 300 mg of LN19184 may improve aspects of cognitive function, elevate BDNF levels, and enhance sleep quality. However, several potential limitations should be considered when interpreting these results. This study was a proof-of-concept trial, designed to generate the first empirical data in support of human cognitive benefits from this novel commercial blend of *T. chebula* and *B. serrata*. Independent replication of these findings is required to establish fully the potential efficacy of LN19184. Additionally, further research is needed to explore the possible mechanisms behind the measured effects of LN19184. There was no direct evaluation of oxidative status, inflammatory markers, amyrins, ellagic acid, gallic acid, acetylcholine, or acetylcholine signaling in the supplemented participants. Future research involving LN19184 should include an assessment of AChE as an indirect indicator of cholinergic function, as both amyrins and boswellic acids can prevent acetylcholine breakdown via AChE inhibition (64). The study population in this study was predominantly male, and information on menopausal status was not collected for the female participants. Conducting future research on LN19184 with a cohort of menopausal women would be of great value, considering that poor memory and sleep are common complaints in this population, and alleviating these symptoms is a major unmet need (78). This study was conducted in a population of adults between the ages of 40–65, and it is unknown if the benefits reported here would extend to an older population with a heightened risk of age-related cognitive decline. Future research should emphasize a greater inclusion of women, older subjects, and additional cognitive tasks.

This proof-of-concept study demonstrated evidence of improved cognitive function, subjective sleep quality, and peripheral BDNF levels among healthy, aging participants supplemented with a novel herbal combination of *T. chebula* and *B. serrata* for 120 days. Improved measures of cognitive and sleep functions were observed as early as 15 days after initiating supplementation, with continued improvements apparent over time. The herbal blend, LN19184, appeared to be well-tolerated in this study population with no serious adverse events or abnormal laboratory values. These findings suggest that supplementation of LN19184 may be beneficial for individuals concerned about cognitive decline associated with aging.

## Data Availability

The raw data supporting the conclusions of this article will be made available by the authors, without undue reservation.
