# Supplementary material for: A standardized combination of Boswellia serrata and Terminalia chebula extracts to improve cognition in adults with subjective memory complaints: a randomized controlled proof-of-concept study
Source: Front Nutr. 2025 Dec 8;12:1695341. doi: 10.3389/fnut.2025.1695341 (PMC12719083; doi:10.3389/fnut.2025.1695341)
Supplement: Supplementary file 1 [file Table_1.DOCX]

| **Parameter** | **Evaluation**  **day** | **Placebo**  **(*N* = 50)** | **LN19184**  **(*N* = 50)** |
| --- | --- | --- | --- |
|  |  |  |  |
| WBC (cells/µL) | Screening | 8.0 ± 1.7 | 8.0 ± 2.2 |
|  | Day 120 | 7.9 ± 1.2 | 7.7 ± 1.7 |
| RBC (10^3^/µL) | Screening | 4.6 ± 0.7 | 4.6 ± 0.7 |
|  | Day 120 | 4.6 ± 0.7 | 4.7 ± 0.6 |
| Hemoglobin (g/dL) | Screening | 13.2 ± 1.4 | 13.3 ± 1.2 |
|  | Day 120 | 13.6 ± 1.7 | 13.7 ± 1.6 |
| Hematocrit (%) | Screening | 40.8 ± 5.3 | 40.8 ± 4.3 |
|  | Day 120 | 42.0 ± 3.9 | 42.3 ± 4.4 |
| Platelet (10^3^/µL) | Screening | 207.0 ± 64.7 | 181.6 ± 46.4 |
|  | Day 120 | 205.1 ± 58.2 | 183.2 ± 40.8 |
| Neutrophils (%) | Screening | 60.5 ± 8.1 | 58.5 ± 8.5 |
|  | Day 120 | 61.2 ± 5.2 | 59.7 ± 7.1 |
| Lymphocytes (%) | Screening | 30.9 ± 8.0 | 31.1 ± 6.8 |
|  | Day 120 | 30.0 ± 5.2 | 30.3 ± 5.9 |
| Monocytes (%) | Screening | 3.5 ± 0.8 | 3.7 ± 0.8 |
|  | Day 120 | 3.8 ± 0.6 | 4.1 ± 1.2 |
| Eosinophils (%) | Screening | 4.6 ± 3.3 | 6.3 ± 4.7 |
|  | Day 120 | 4.3 ± 1.9 | 5.3 ± 2.7 |
| Basophils (%) | Screening | 0.5 ± 0.3 | 0.5 ± 0.2 |
|  | Day 120 | 0.6 ± 0.1 | 0.6 ± 0.3 |

Table S1: Hematological parameters. Data are presented as means ± standard deviation of patients who completed the study. WBC (white blood cells), RBC (red blood cells).
