# Supplementary material for: A standardized combination of Boswellia serrata and Terminalia chebula extracts to improve cognition in adults with subjective memory complaints: a randomized controlled proof-of-concept study
Source: Front Nutr. 2025 Dec 8;12:1695341. doi: 10.3389/fnut.2025.1695341 (PMC12719083; doi:10.3389/fnut.2025.1695341)
Supplement: Supplementary file 2 [file Table_2.DOCX]

| **Parameter** | **Day** | **Placebo**  **(*N* = 50)** | **LN19184**  **(*N* = 50)** |
| --- | --- | --- | --- |
|  |  |  |  |
| Fasting glucose (mg/dL) | Screening | 84.3 ± 8.8 | 88.5 ± 14.5 |
|  | Day 120 | 89.4 ± 7.2 | 89.5 ± 8.7 |
| Sodium (mEq/L) | Screening | 141.4 ± 5.4 | 140.9 ± 2.9 |
|  | Day 120 | 140.8 ± 4.9 | 141.3 ± 2.4 |
| Potassium (mmol/L) | Screening | 4.7 ± 0.6 | 4.7 ± 0.7 |
|  | Day 120 | 4.7 ± 0.4 | 4.7 ± 0.4 |
| Urea nitrogen (mg/dL) | Screening | 25.7 ± 6.5 | 26.9 ± 6.0 |
|  | Day 120 | 27.8 ± 6.3 | 28.5 ± 6.0 |
| Creatine (mg/dL) | Screening | 0.8 ± 0.3 | 0.7 ± 0.1 |
|  | Day 120 | 0.8 ± 0.2 | 0.8 ± 0.1 |
| Albumin (g/dL) | Screening | 4.6 ± 0.3 | 4.6 ± 0.3 |
|  | Day 120 | 4.5 ± 0.3 | 4.5 ± 0.3 |
| Total bilirubin (mg/dL) | Screening | 0.5 ± 0.3 | 0.5 ± 0.2 |
|  | Day 120 | 0.6 ± 0.2 | 0.6 ± 0.2 |
| ALP (U/L) | Screening | 105.0 ± 30.6 | 111.2 ± 26.9 |
|  | Day 120 | 106.8 ± 26.1 | 113.2 ± 20.1 |
| AST (U/L) | Screening | 30.6 ± 17.0 | 28.0 ± 6.8 |
|  | Day 120 | 27.9 ± 7.9 | 28.0 ± 5.6 |
| ALT (U/L) | Screening | 36.6 ± 33.5 | 33.1 ± 20.7 |
|  | Day 120 | 31.3 ± 18.3 | 32.3 ± 19.7 |
| Total cholesterol (mg/dL) | Screening | 173.8 ± 36.1 | 167.3 ± 32.5 |
|  | Day 120 | 171.7 ± 36.1 | 169.7 ± 29.3 |
| Triglycerides (mg/dL) | Screening | 208.6 ± 122.7 | 203.9 ± 109.7 |
|  | Day 120 | 163.8 ± 66.8 | 174.5 ± 70.1 |
| HDL (mg/dL) | Screening | 42.4 ± 9.0 | 38.7 ± 8.4 |
|  | Day 120 | 43.3 ± 8.3 | 41.6 ± 7.5 |
| LDL (mg/dL) | Screening | 91.5 ± 29.0 | 87.1 ± 24.0 |
|  | Day 120 | 92.3 ± 28.1 | 89.6 ± 21.2 |

Supplemental Table S2: Serum biochemistry parameters. Data are presented as mean ± standard deviation of patients who completed the study. ALP (alkaline phosphatase), ALT (alanine transaminase), AST (aspartate aminotransferase), HDL (high-density lipoprotein), LDL (low-density lipoprotein).
