# Supplementary material for: A standardized combination of Boswellia serrata and Terminalia chebula extracts to improve cognition in adults with subjective memory complaints: a randomized controlled proof-of-concept study
Source: Front Nutr. 2025 Dec 8;12:1695341. doi: 10.3389/fnut.2025.1695341 (PMC12719083; doi:10.3389/fnut.2025.1695341)
Supplement: Supplementary file 3 [file Table_3.DOCX]

**Table S3: Effect, effect size, within- and between-group comparisons of CANTAB tests presented in Figure 3**

| **Parameter** | **Group** | **Evaluation days** | | | | | |  |
| --- | --- | --- | --- | --- | --- | --- | --- | --- |
|  |  | **Day 1**  **(Baseline)** | **Day 15** | **Day 30** | **Day 60** | **Day 90** | **Day 120** | Main Effect *p*-value  Effect Size (ɳ^2^) |
| **Rapid Visual Information**  **Processing Latency (ms)** | LN19184±SD | 725.7±312.2 | 688.3±276.0 | 622.9±213.3 | 609.5±208.2 | 580.3±150.6 | 541.7±141.9* | (t) *p*=0.001^#^ ɳ^2^=0.053 (S)  (trt) *p*=0.04^#^ ɳ^2^=0.053 (S)  (int) *p*=0.02^#^ ɳ^2^=0.036 (S) |
|  | PLA±SD | 762.9±325.5 | 665.8±210.8 | 631.6±209.6 | 702.3±237.4 | 737.1 ± 231.8 | 689.4 ± 199.3 |  |
|  | MD±SE | -37.2±71.3 | 22.5±55.1 | -8.7±47.3 | -92.8±49.9 | -156.8 ± 43.5^ | -147.7 ± 38.5^ |  |
|  | 95% CI | -179.1, 104.8 | -87.2, 132.2 | -102.8, 85.5 | -192.0, 6.5 | -243.3, -70.2 | -224.4, -71.0 |  |
| **Delayed Match to Sample Latency (ms)** | LN19184±SD | 3689.2±3095.5 | 4120.5±3490.3 | 4596.3±6168.0 | 2702.0±1413.4 | 2625.7±1539.6 | 2755.7±2526.5 | (t) *p*=0.13  (trt) *p*=0.11  (int) *p*=0.036^#^ ɳ^2^=0.033 (S) |
|  | PLA±SD | 3697.7±3129.0 | 4523.7±4821.8 | 4183.7±4033.5 | 3570.1±2454.7 | 5345.8±5414.3 | 4416.7±4224.8 |  |
|  | MD±SE | -8.5±696. 1 | -403.3±937.7 | 412.6±1171. 6 | -868.2±445.1 | -2720.1±880.5**^** | -1661.0±773.8**^** |  |
|  | 95% CI | -1394.3,1377.2 | -2270.1, 1463.5 | -1919.8, 2745.1 | -1754.3, 18.0 | -4473.1, -967. 1 | -3201.6, -120.4 |  |
| **Multitasking Latency**  **(ms)** | LN19184±SD | 817.0±224.0 | 786.8±167.1 | 804.3±217.2 | 749.5±205.2 | 672.0 ± 167.1***** | 653.5 ± 214.7 | (t) *p*=0.444  (trt) *p*<0.001^#^ ɳ^2^=0.183 (L)  (int) *p*<0.001^#^ ɳ^2^=0.124 (M) |
|  | PLA±SD | 843.6±227.9 | 751.2±130.9 | 824.3±237.3 | 876.4±237.0 | 972.4 ± 279.1 | 955.0 ± 322.2 |  |
|  | MD±SE | -26.6±50.5 | 35.7±33.7 | -19.9±50.8 | -126.9±49.5^ | -300.4 ± 51.1**^** | -301.5 ± 60.9**^** |  |
|  | 95% CI | -127.2, 74.0 | -31.4, 102.7 | -121.1, 81.2 | -225.5, -28.4 | -402.2, -198.6 | -422.8, -180.2 |  |
| **Spatial Working Memory Errors (#)** | LN19184±SD | 23.8±4.4 | 21.4±8.8 | 18.8±10.7* | 20.4±9.5 | 19.8±10.6 | 16.9 ±11.4***** | (t) *p*=0.018^#^ ɳ^2^ =0.035 (S)  (trt) *p*=0.015^#^ ɳ^2^=0.074 (M)  (int) *p*=0.034^#^ ɳ^2^ = 0.030 (S) |
|  | PLA±SD | 22.7±5.4 | 23.4±7.6 | 21.8±7.4 | 24.3±8.1 | 23.9 ±6.7 | 23.0 ±6.1 |  |
|  | MD±SE | 1.1±1.1 | -2.0±1.8 | -3.1±2.1 | -3.9±2.0**^** | -4.1±2.0**^** | -6.1±2.1**^** |  |
|  | 95% CI | -1.1, 3.3 | -5.7, 1.6 | -7.2, 1.0 | -7.9, -0.0 | -8.1, -0.1 | -10.2, -2.0 |  |
| **Multitasking**  **Total Correct** (#) | LN19184±SD | 105.1±31.8 | 108.9±24.1 | 116.8±25.5 | 120.5±23.6***** | 132.5±21.2***** | 128.2±27.2***** | (t) *p*<0.001^#^ ɳ^2^ =0.090 (M)  (trt) *p*=0.454  (int) *p*<0.001^#^ ɳ^2^ =0.058 (S) |
|  | PLA±SD | 108.7±29.1 | 116.6±28.9 | 114.0±26.3 | 123.9±27.7***** | 115.4±28.2 | 113.6±34.2 |  |
|  | MD±SE | -3.6±6.8 | -7.7±5.9 | 2.8±5.8 | -3.4±5.7 | 17.1±5.6^ | 14.7±6.9^ |  |
|  | 95% CI | -17.2, 10.0 | -19.5, 4.1 | -8.7, 14.3 | -14.9, 8.0 | 6.0, 28.1 | 1.0, 28.4 |  |
| **Delayed Match to Sample**  **(% Correct)** | LN19184±SD | 42.1±17.5 | 47.4±17.4 | 45.7±13.6 | 50.6±14.4 | 50.1±15.7 | 52.3±15.9 | (t) *p*=0.051  (trt) *p*=0.08  (int) *p*=0.021^#^ ɳ^2^ =0.035 (S) |
|  | PLA±SD | 42.4±16.3 | 46.4±14.3 | 46.2±17.1 | 48.9±16.8 | 45.3±17.7 | 38.2±16.1 |  |
|  | MD±SE | -0.4±3.8 | 1.0±3.6 | -0.4±3.4 | 1.8±3.5 | 4.9±3.7 | 14.1±3.6**^** |  |
|  | 95% CI | -7.9, 7.2 | -6.1, 8.1 | -7.3, 6.4 | -5.2, 8.7 | -2.6, 12.3 | 7.0, 21.2 |  |
| **Spatial Working**  **Memory Strategy**  **(Score)** | LN19184±SD | 9.6±1.4 | 9.5±1.6 | 9.1±2.5 | 8.7±2.1 | 8.5±2.5 | 8.2±2.0***** | (t) *p*=0.022^#^ ɳ^2^ =0.035 (S)  (trt) *p*=0.031^#^ ɳ^2^ =0.058 (S)  (int) *p*=0.48 |
|  | PLA±SD | 9.6±1.1 | 9.4±1.5 | 9.5±1.5 | 9.4±2.3 | 9.0±2.1 | 9.2±2.3 |  |
|  | MD±SE | 0.0±0.3 | 0.1±0.3 | -0.5±0.5 | -0.7±0.5 | -0.5±0.5 | -1.0±0.5**^** |  |
|  | 95% CI | -0.5, 0.6 | -0.6, 0.8 | -1.4, 0.5 | -1.7, 0.3 | -1.6, 0.5 | -1.9, 0.0 |  |
| **Paired Associates Learning Total Errors (#)** | LN19184±SD | 47.6±15.3 | 42.4±16.9 | 45.7±16.7 | 39.7±17.3 | 29.0±19.9***** | 25.6±19.5***** | (t) *p*<0.001^#^ ɳ^2^ =0.111 (M)  (trt) *p*=0.029^#^ ɳ^2^ =0.060 (M)  (int) *p*<0.001^#^ ɳ^2^ =0.058 (S) |
|  | PLA±SD | 46.6±14.8 | 43.3±18.8 | 46.5±17.1 | 38.6±17.3 | 44.3±18.3 | 40.1±18.3 |  |
|  | MD±SE | 1.0±3.4 | -0.8±4.0 | -0.8±3.8 | 1.2±3.9 | -15.3±4.3**^** | -14.5±4.2**^** |  |
|  | 95% CI | -5.7, 7.7 | -8.8, 7.1 | -8.3, 6.7 | -6.5, 8.9 | -23.8, -6.8 | -22.9, -6.1 |  |
| **Paired Associate Learning- 1^st^ Attempt Memory**  **(Score)** | LN19184±SD | 4.9±3.4 | 6.2±3.8 | 5.4±3.8 | 6.3±3.6 | 9.2±5.4* | 10.2±5.7* | (t) *p*<0.001^#^ ɳ^2^ =0.085 (M)  (trt) *p*=0.014^#^ ɳ^2^ =0.074 (M)  (int) *p*<0.001^#^ ɳ^2^ =0.061 (M) |
|  | PLA±SD | 5.2±3.5 | 6.1±4.9 | 5.4±4.3 | 6.8±4.0 | 5.3±3.8 | 5.6±4.1 |  |
|  | MD±SE | -0.3±0.8 | 0.1±1.0 | -0.0±0.9 | -0.5±0.8 | -3.9±1.1**^** | -3.7±1.1**^** |  |
|  | 95% CI | -1.8, 1.2 | -1.2, 2.1 | -1.8, 1.8 | -2.2, 1.2 | 1.8, 6.0 | 1.4, 5.9 |  |

Data presented as mean ± standard deviation (SD) or mean difference (MD) ± standard error (SE) and 95% Confidence Interval (95% CI). Significance is considered *p*<0.05 after mixed factorial repeated measure ANOVA adjusted with Bonferroni correction for multiple comparisons. * Indicates within-group significance (vs. baseline), ^ indicates significant difference between group means (LN19184 vs. placebo), # indicates significant main effect of time (t), treatment (trt), or time x treatment interaction (int). Partial effect size (ɳ^2^) is defined as small (S), ≥ 0.02 <0.6; moderate (M), ≥ 0.06 <0.14; and large, ≥ 0.14 (L). LN19184 n=41, PLA (Placebo) n=39. CANTAB (Cambridge Neuropsychological Test Automated Battery).
