# Supplementary material for: A standardized combination of Boswellia serrata and Terminalia chebula extracts to improve cognition in adults with subjective memory complaints: a randomized controlled proof-of-concept study
Source: Front Nutr. 2025 Dec 8;12:1695341. doi: 10.3389/fnut.2025.1695341 (PMC12719083; doi:10.3389/fnut.2025.1695341)
Supplement: Supplementary file 4 [file Table_4.DOCX]

**Table S4: Effect, effect size, within- and between-group comparisons of additional CANTAB tests**

| **Parameter** | **Group** | **Evaluation days** | | | | | |  |
| --- | --- | --- | --- | --- | --- | --- | --- | --- |
|  |  | **Day 1**  **(Baseline)** | **Day 15** | **Day 30** | **Day 60** | **Day 90** | **Day 120** | Main Effect p-value Effect Size (ɳ^2^) |
| **Delayed Match to Sample, Probability of Error (%)** | LN19184±SD | 0.52±0.23 | 0.48±0.22 | 0.47±0.22 | 0.44±0.20 | 0.43±0.22 | 0.44±0.22 | (t) *p*=0.027**^#^** ɳ^2^=0.032 (S)  (trt) *p*=0.117 ɳ^2^=0.024 (S)  (int) *p*=0.097 ɳ^2^=0.032 (S) |
|  | PLA±SD | 0.52±0.25 | 0.49±0.21 | 0.52±0.22 | 0.42±0.23 | 0.48±0.23 | 0.60±0.19 |  |
|  | MD±SE | 0.00±0.54 | -0.01±0.05 | -0.05±0.05 | -0.02±0.05 | -0.05±0.05 | -0.16±0.05**^** |  |
|  | 95% CI | -0.10, 0.11 | -0.10, 0.09 | -0.15, 0.15 | -0.08, 0.11 | -0.15, 0.05 | -0.25, -0.07 |  |
| **Spatial Span, Forward Span Length (#)** | LN19184±SD | 2.6±1.7 | 2.6±1.9 | 2.6±1.7 | 3.1±1.8 | 3.5±2.2 | 3.9±1.9***** | (t) *p*<0.001**^#^** ɳ^2^=0.058 (S)  (trt) *p*=0.865  (int) *p*=0.540 |
|  | PLA±SD | 2.5±1.4 | 3.0±2.0 | 2.9±1.7 | 3.3±2.0 | 3.3±2.3 | 3.4±2.2 |  |
|  | MD±SE | 0.1±0.4 | -0.4±0.4 | -0.3±0.4 | -0.2±0.4 | 0.1±0.5 | 0.5±0.5 |  |
|  | 95% CI | -0.6, 0.8 | -1.3, 0.4 | -1.1, 0.4 | -1.1, 0.6 | -0.9, 1.1 | -0.4, 1.4 |  |
| **Spatial Span,**  **Total Usage Errors (#)** | LN19184±SD | 3.6±2.7 | 3.4±2.4 | 3.2±2.4 | 3.1±2.5 | 1.9±2.0 | 2.5±2.6 | (t) *p*= 0.969  (trt) *p*=0.106 ɳ^2^= 0.033 (S)  (int) *p=*0.005**^#^** ɳ^2^= 0.04 (S) |
|  | PLA±SD | 3.0±2.3 | 2.9±2.7 | 3.4±3.4 | 3.2±2.8 | 4.1±2.9 | 3.9±3.1 |  |
|  | MD±SE | 0.7±0.6 | 0.5±0.6 | -0.2±0.7 | -0.2±0.6 | -2.1±0.6^ | -1.4±0.6^ |  |
|  | 95% CI | -0.5, 1.8 | -0.6, 1.6 | -1.6, 1.1 | -1.4, 1.0 | -3.3, -1.0 | -2.7, -0.2 |  |
| **Spatial Span, Forward Attempts (#)** | LN19184±SD | 1.6±0.5 | 1.6±0.5 | 1.6±0.6 | 1.5±0.6 | 1.4±0.6 | 1.2±0.4* | (t) *p*=0.165  (trt) *p*=0.140 ɳ^2^ = 0.029 (S)  (int) *p*<0.001**^#^** ɳ^2^ = 0.054 (S) |
|  | PLA±SD | 1.5±0.6 | 1.5±0.6 | 1.7±0.6 | 1.6±0.6 | 1.6±0.6 | 1.7±0.6 |  |
|  | MD±SE | 0.1±0.1 | 0.2±0.1 | -0.2±0.1 | -0.1±0.2 | -0.2±0.1 | -0.5±0.1^ |  |
|  | 95% CI | -0.1, 0.4 | -0.1, 0.4 | -0.4, 0.1 | -0.4, 0.2 | -0.5, 0.1 | -0.8, -0.3 |  |
| **Simple Reaction Time, Mean Time (ms)** | LN19184±SD | 593.8±265.2 | 541.6±198.4 | 595.3±222.7 | 610.3±259.5 | 608.7±262.1 | 546.1±249.3 | (t) *p*=0.131 ɳ^2^ = 0.021 (S)  (trt) *p*=0.200 ɳ^2^ = 0.021 (S)  (int) *p*=0.143 ɳ^2^ = 0.021 (S) |
|  | PLA±SD | 621.4±360.8 | 570.8±370.1 | 624.5±338.9 | 624.4±338.9 | 680.0±211.8 | 719.7±278.0 |  |
|  | MD±SE | -27.6±70.6 | -29.3±66.1 | -29.2±63.8 | -14.1±54.7 | -71.3±53.4 | -173.5±59.0**^** |  |
|  | 95% CI | -168.1, 112.8 | -160.8, 102.2 | -156.2, 97.9 | -122.9, 94.7 | -177.7, 35.1 | -291.0, -56.1 |  |
| **Reaction Time,**  **5-Choice (ms)** | LN19184±SD | 600.3±430.6 | 600.1±280.9 | 621.6±304.3 | 559.7±184.9 | 583.9±215.8 | 621.6±430.9 | (t) *p*=0.040**^#^** ɳ^2^=0.034 (S)  (trt) *p*=0.518  (int) *p*=0.071 ɳ^2^=0.026 (S) |
|  | PLA±SD | 570.1±233.4 | 596.9±260.2 | 573.4±209.8 | 569.9±164.7 | 707.5±290.7 | 737.6±269.1 |  |
|  | MD±SE | 30.1±78.0 | 3.2±60.6 | 48.1±58.7 | -10.2±39.2 | -123.6±57.1**^** | -116.0±80.8 |  |
|  | 95% CI | -125.2, 185.5 | -117.5, 123.9 | -68.8, 165.1 | -88.3, 67.9 | -237.1, -10.0 | -276.9, 44.8 |  |
| **Reaction Time,**  **5-Choice**  **Movement (ms)** | LN19184±SD | 1235.8±1199.0 | 926.4±860.0 | 902.3±794.1 | 930.1±945.7 | 978.5±905.2 | 942.6±1003.0 | (t) *p*=0.002**^#^** ɳ^2^ = 0.055 (S)  (trt) *p*=0.700  (int) *p*=0.014**^#^** ɳ^2^ = 0.041 (S) |
|  | PLA±SD | 1090.1±1088.4 | 653.4±577.4 | 751.7±750.1 | 1158.4±1060.6 | 1212.4±941.2 | 1396.7±767.6 |  |
|  | MD±SE | 145.7±256.4 | 273.0±164.6 | 150.6±172.9 | -228.3±224.4 | -233.6±206.4 | -454.1±200.4**^** |  |
|  | 95% CI | -364.8, 656.1 | -54.7, 600.7 | -193.6, 494.8 | -675.1, 218.5 | -644.6, 177.4 | -853.1, -55.8 |  |
| **Paired Associates Learning, Total Attempts (#)** | LN19184±SD | 7.2±2.4 | 7.5±2.1 | 7.4±2.4 | 7.8±2.0 | 8.1±2.6 | 7.6±2.2 | (t) *p*< 0.043**^#^** ɳ^2^ = 0.029 (S)  (trt) *p*= 0.949  (int) *p=*0.968 |
|  | PLA±SD | 7.2±2.2 | 7.5±2.4 | 7.1±2.2 | 7.8±2.2 | 8.0±3.0 | 7.9±2.0 |  |
|  | MD±SE | 0.0±0.5 | 0.0±0.5 | 0.3±0.5 | 0.0±0.5 | 0.1±0.6 | -0.3±0.5 |  |
|  | 95% CI | -1.0, 1.0 | -0.1, 0.1 | -0.7, 1.3 | -0.9, 1.0 | -1.2, 1.3 | -1.2, 0.6 |  |
| **Multitasking,**  **Total Incorrect (#)** | LN19184±SD | 27.6±13.2 | 25.1±17.3 | 18.9±12.0 | 18.5±17.0 | 14.3±13.5***** | 13.1±12.7***** | (t) *p*=0.017**^#^** ɳ^2^=0.037 (S)  (trt) *p*=0.033**^#^** ɳ^2^=0.057 (S)  (int) *p*=0.006**^#^** ɳ^2^=0.044 (S) |
|  | PLA±SD | 24.1±15.7 | 20.5±12.8 | 24.6±14.3 | 18.5±19.6 | 20.8±19.3 | 25.8±19.4 |  |
|  | MD±SE | 3.5±3.2 | 4.6±3.4 | -6.5±2.9^ | -0.3±5.0 | -6.5±3.7 | -12.7±3.6**^** |  |
|  | 95% CI | -2.9, 10.0 | -2.2, 11.4 | -12.4, -0.7 | -8.8, 8.1 | -13.9, 0.8 | -20.0, -5.5 |  |
| **Multitasking,**  **Incongruency Cost (ms)** | LN19184±SD | 41.5±62.1 | 49.2±87.3 | 59.2±60.8 | 63.4±82.6 | 56.0±51.6 | 42.0±69.9 | (t) *p*=0.360  (trt) *p*=0.826  (int) *p*=0.392 |
|  | PLA±SD | 64.2±75.3 | 55.7±80.1 | 36.2±63.3 | 66.7±77.4 | 41.5±73.4 | 37.6±70.4 |  |
|  | MD±SE | -22.7±15.4 | -6.6±18.7 | 22.9±13.9 | -3.3±17.9 | 14.9±14.1 | 4.4±15.7 |  |
|  | 95% CI | -53.8, 7.9 | -43.9, 30.8 | -4.7, 50.6 | -38.9, 32.3 | -13.6, 42.6 | -26.9, 35.6 |  |
| **Multitasking, Cost (ms)** | LN19184±SD | -39.3±161.1 | -24.3±166.5 | -12.3±157.5 | 54.4±116.9 | -14.6±118.4 | 4.9±120.2 | (t) *p*=0.248  (trt) *p*= 0.478  (int) *p*=0.038**^#^** ɳ^2^=0.031 (S) |
|  | PLA±SD | -51.7±169.9 | 16.3±137.4 | 23.3±121.8 | -44.0±135.2 | -4.3±175.6 | -28.2±165.9 |  |
|  | MD±SE | 12.4±37.0 | -40.5±34.2 | -35.5±31.6 | 98.5±28.2^ | -10.3±33.3 | 33.1±32.3 |  |
|  | 95% CI | -61.3, 86.1 | -108.7, 27.6 | -98.4, 27.4 | 42.4, 154.6 | -76.6, 56.1 | -31.1, 97.4 |  |
| **Match to Sample, Time to All Correct (median, ms)** | LN19184±SD | 7972.8±7457.1 | 6113.2±5763.5 | 6310.0±6264.3***** | 4255.6±3019.0***** | 3785.2±2883.3***** | 4182.7±5833.4***** | (t) *p*<0.001**^#^** ɳ^2^=0.078 (M)  (trt) *p*= 0.525  (int) *p*=0.048**^#^** ɳ^2^=0.032 (S) |
|  | PLA±SD | 6257.0±5561.3 | 5320.9±4570.2 | 7952.4±7827.9 | 5227.4±3972.0 | 5498.1±3609.9 | 5600.4±3154.2 |  |
|  | MD±SE | 1715.9±1483.0 | 792.3±1172.2 | -1642.5±1593.1 | -971.8±792.5 | -1712.9±734.1^ | -1417.8±1059.0 |  |
|  | 95% CI | -1237.2, 4668.9 | -1541.8, 3126.4 | -4815.0, 1529.5 | -2549.9, 606.3 | -3174.7, -251.1 | -3526.5, 690.9 |  |
| **Match to Sample, Time to All Correct (mean, ms)** | LN19184±SD | 9877.9±9101.7 | 8660.7±8134.0 | 8526.7±7862.1 | 5934.7±4372.7***** | 5115.5±3820.4***** | 5847.8±7358.7 | (t) *p*<0.001**^#^** ɳ^2^=0.089 (M)  (trt) *p*= 0.660  (int) *p*=0.154 ɳ^2^=0.021(S) |
|  | PLA±SD | 8265.0±7013.5 | 7757.1±6536.7 | 10289.7±9858.8 | 6819.3±5482.6 | 6814.5±4197.2 | 7068.9±4347.5 |  |
|  | MD±SE | 1612.9±1823.3 | 903.6±1655.0 | -1763.0±1988.7 | -884.6±1106.0 | -1699.0±896.6 | -1222.1±1360.2 |  |
|  | 95% CI | -2016.9, 5242.8 | -2391.2, 4198.4 | -5722.2, 2196.2 | -3086.4, 1317.3 | -3484.0, 85.9 | -3930.0, 1485.8 |  |

Data presented as mean ± standard deviation (SD) or mean difference (MD) ± standard error (SE) and 95% Confidence Interval (95% CI). Significance is considered *p*<0.05 after mixed factorial repeated measure ANOVA adjusted with Bonferroni correction for multiple comparisons. * Indicates within-group significance (vs. baseline), ^ indicates significant difference between group means (LN19184 vs. placebo), # indicates significant main effect of time (t), treatment (trt), or time x treatment interaction (int). Partial effect size (ɳ^2^) is defined as small (S), ≥ 0.02 <0.6; moderate (M), ≥ 0.06 <0.14; and large, ≥ 0.14 (L). LN19184 n=41, PLA (Placebo) n=39. CANTAB (Cambridge Neuropsychological Test Automated Battery).
